# *KCNJ3* is a new independent prognostic marker for estrogen receptor positive breast cancer patients

**DOI:** 10.18632/oncotarget.13224

**Published:** 2016-11-08

**Authors:** Sarah Kammerer, Armin Sokolowski, Hubert Hackl, Dieter Platzer, Stephan Wenzel Jahn, Amin El-Heliebi, Daniela Schwarzenbacher, Verena Stiegelbauer, Martin Pichler, Simin Rezania, Heidelinde Fiegl, Florentia Peintinger, Peter Regitnig, Gerald Hoefler, Wolfgang Schreibmayer, Thomas Bauernhofer

**Affiliations:** ^1^ Molecular Physiology Group, Institute of Biophysics, Medical University of Graz, Austria; ^2^ Research Unit on Ion Channels and Cancer Biology, Medical University of Graz, Austria; ^3^ Division of Bioinformatics, Biocenter, Medical University of Innsbruck, Austria; ^4^ Institute of Pathology, Medical University of Graz, Austria; ^5^ Institute of Cell Biology, Histology and Embryology, Medical University of Graz, Austria; ^6^ Division of Oncology, Department of Internal Medicine, Medical University of Graz, Austria; ^7^ Department of Experimental Therapeutics, The University of Texas MD Anderson Cancer Center, Houston, TX, USA; ^8^ Department of Gynecology and Obstetrics, Medical University of Innsbruck, Austria; ^9^ Present address: Division of Prosthodontics, Restorative Dentistry, Periodontology and Implantology, Medical University of Graz, Austria

**Keywords:** KCNJ3, GIRK1, biomarker, estrogen receptor positive breast cancer, RNA in situ hybridization

## Abstract

Numerous studies showed abnormal expression of ion channels in different cancer types. Amongst these, the potassium channel gene *KCNJ3* (encoding for GIRK1 proteins) has been reported to be upregulated in tumors of patients with breast cancer and to correlate with positive lymph node status. We aimed to study *KCNJ3* levels in different breast cancer subtypes using gene expression data from the TCGA, to validate our findings using RNA *in situ* hybridization in a validation cohort (GEO ID GSE17705), and to study the prognostic value of *KCNJ3* using survival analysis. In a total of > 1000 breast cancer patients of two independent data sets we showed a) that *KCNJ3* expression is upregulated in tumor tissue compared to corresponding normal tissue (*p* < 0.001), b) that *KCNJ3* expression is associated with estrogen receptor (ER) positive tumors (*p* < 0.001), but that *KCNJ3* expression is variable within this group, and c) that ER positive patients with high *KCNJ3* levels have worse overall (*p* < 0.05) and disease free survival probabilities (*p* < 0.01), whereby *KCNJ3* is an independent prognostic factor (*p* <0.05). In conclusion, our data suggest that patients with ER positive breast cancer might be stratified into high risk and low risk groups based on the *KCNJ3* levels in the tumor.

## INTRODUCTION

Human G-protein activated inward rectifier potassium channel subunits (GIRKs) are encoded by four genes (*KCNJ3; KCNJ5; KCNJ6; KCNJ9)*. GIRK1-4 proteins form homo- or hetero-tetrameric ion channels, function as G-protein effectors in the plasma membrane and thereby regulate cellular excitability and activity via neurotransmitters and hormones [1]. GIRKs are involved in the regulation of functions as diverse as heartbeat, reward mechanisms, learning and memory functions, insulin secretion, blood platelet aggregation and lipid metabolism [1–6]. Increasing evidence suggests an involvement of genes encoding for GIRKs in tumorigenesis and tumor growth. Benign adenomas of adrenal cortex cells, which lead to aldosteronism and severe hypertension, have been linked to somatic mutations in the *KCNJ9* gene encoding GIRK4 [7, 8]. Upregulation of *KCNJ3* gene products (i.e. GIRK1 mRNA and protein; synonyms: KGA, Kir3.1) was reported for non-small cell lung cancer [9] and pancreatic adenocarcinomas [10]. Correlation of increased *KCNJ3* expression levels and breast cancer progression has been shown by several studies: Stringer et al. [11] reported increased levels of *KCNJ3* mRNA in primary invasive breast carcinomas when compared to corresponding normal breast tissue and found a positive correlation between *KCNJ3* mRNA expression levels in the tumor and the number of metastatic lymph nodes. Brevet et al. [12] confirmed on protein level that GIRK1 expression is higher in breast tumors than in normal breast tissue. Functional roles of *KCNJ3* expression in breast cancer were investigated by Rezania et al. [13], who demonstrated that stable overexpression of *KCNJ3* in MCF-7 breast cancer cells results in increased motility, invasiveness and angiogenesis compared to controls. Based on these results, we intended to study and validate *KCNJ3* expression in invasive breast carcinoma samples as potential new prognostic biomarker. Consequently, the aim of the current study was a) to compare *KCNJ3* expression levels between breast tumors and surrounding normal breast tissue, b) to screen the large patient cohort of The Cancer Genome Atlas (TCGA) for differential expression of *KCNJ3* in clinically relevant subsets of breast cancer patients, c) to perform overall and disease free survival analysis to retrieve any possible prognostic value of *KCNJ3* for breast cancer patients, d) to validate TCGA data by RNA *in situ* hybridization on formalin-fixed, paraffin-embedded (FFPE) breast cancer tissue samples of a previously characterized cohort (GEO ID GSE17705, [14]), and e) to get insight into the effects of *KCNJ3* upregulation by performing mammosphere formation assays with MCF-7 breast cancer cell lines overexpressing *KCNJ3*. Our results suggest that *KCNJ3* upregulation is an independent prognostic factor for estrogen receptor positive breast cancer.

## RESULTS

### *KCNJ3* expression is upregulated in breast tumors compared to normal breast tissue

**Figure 1:**
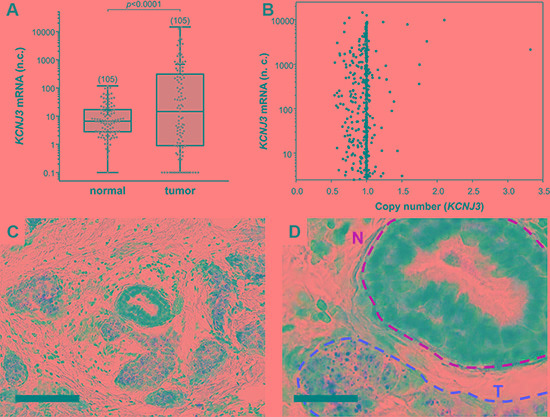
*KCNJ3* mRNA levels are higher in tumor than in surrounding normal tissue (**A**) *KCNJ3* mRNA expression in normal and corresponding tumor tissue of breast cancer patients of the TCGA. *Whiskers represent the lowest and the highest* value, *single data points are shown by grey circles, the black* + *marks the mean* value. *Number of samples analysed is given in brackets above each box. A Wilcoxon signed rank* test *was used for analysis (p < 0.001); n.c.: normalized counts*. (**B**) *KCNJ3* gene copy numbers versus *KCNJ3* mRNA expression of breast cancer patients of the TCGA (*n* = 890). *A copy number of 1.0 indicates that no gene amplification or deletion on DNA level has happened. Spearman rank correlation analysis revealed no statistical significant increase or decrease in KCNJ3 gene copy number* (*r*S*= -0.0207; not significant); n.c.: normalized counts.* (**C**) *KCNJ3* RNA *in situ* hybridization of a patient sample with high *KCNJ3* expression. *Positive signals (brown spots) are present in tumor cells only. Scale bar: 100 μm.* (**D**) Detail of (C). *N (yellow): normal duct; T (red): tumor area.* *Scale bar: 25 μm.*

First, we investigated whether *KCNJ3* mRNA expression is higher in breast tumors when compared to corresponding normal tissue. Analysis of TCGA gene expression data of 105 tumor samples with corresponding normal breast samples showed significantly higher *KCNJ3* mRNA levels in the tumors when compared to normal breast tissue (median 14.6 vs. 6.6 normalized counts; *p* < 0.001; Figure 1A).

Upregulation of mRNAs in tumor cells might be caused by different mechanisms, including gene locus amplification on DNA level [15–17]. To explore gene amplification as a possible cause for the observed increase in *KCNJ3* mRNA expression levels in breast tumors, we studied the gene copy numbers of 890 TCGA patient samples. Figure 1B shows that only two patients had a gene locus amplification (*KCNJ3* copy number > 2) that resulted in increased mRNA expression. Overall, and despite a broad range of *KCNJ3* mRNA expression levels, there was no significant increase or decrease in gene copy number (Spearman rank correlation coefficient *r*_S_: −0.02; *p* = 0.536), suggesting that high mRNA levels are generated by other mechanisms than copy number variations.

In order to validate *KCNJ3* expression levels in tumor and normal cells, RNA *in situ* hybridization (ISH) was performed on breast cancer tissue samples to locate *KCNJ3* expression in cancerous as well as in surrounding tumor stroma and normal breast epithelial cells. The results proved that *KCNJ3* expression is present in tumor cells, but not in non-neoplastic cells including normal mammary ducts (Figure 1C and 1D).

### *KCNJ3* expression is associated with estrogen receptor positive tumors

**Figure 2:**
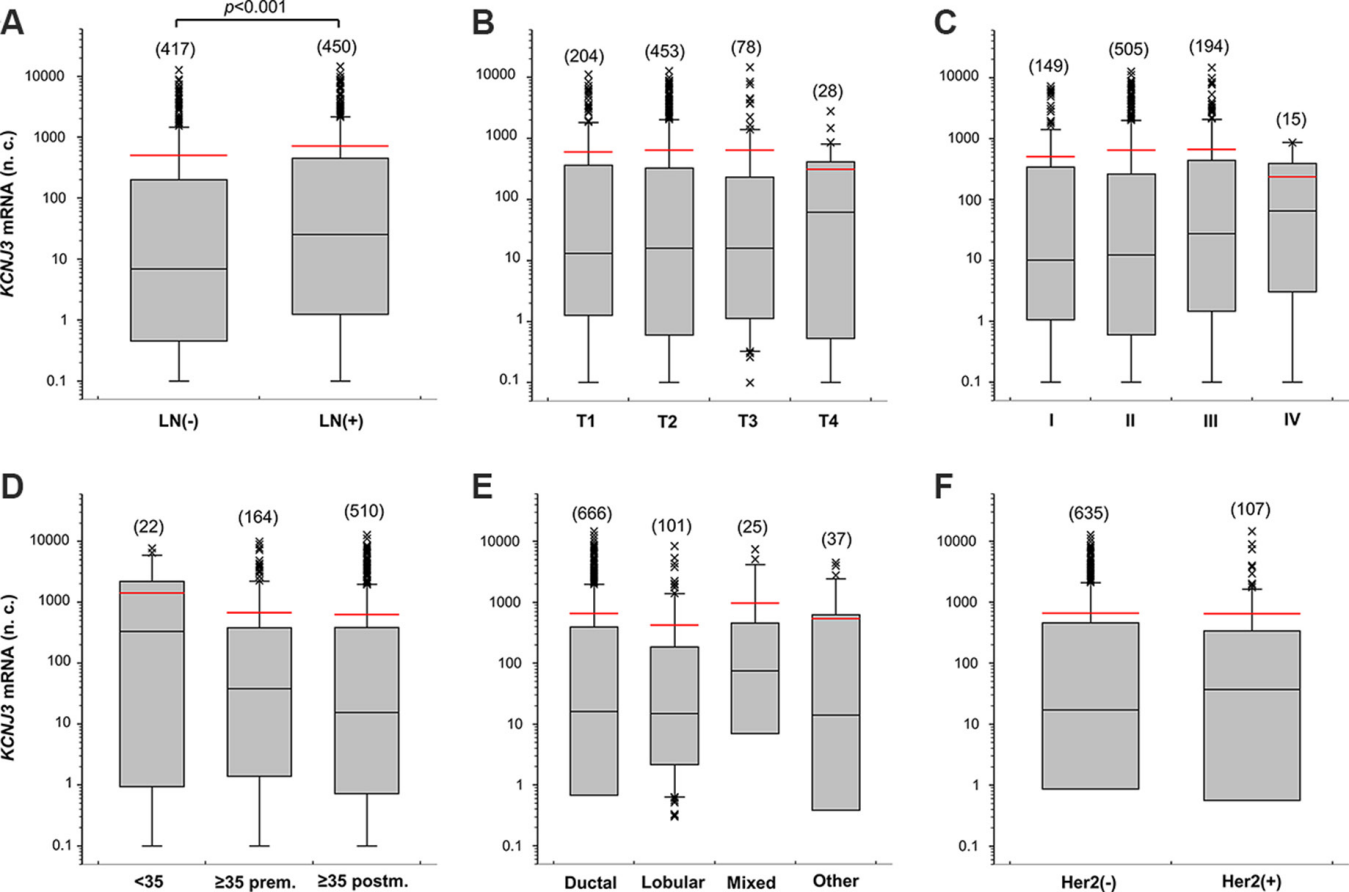
*KCNJ3* mRNA levels in different subsets of breast cancer patients of the TCGA *KCNJ3* mRNA expression levels in breast cancer patient samples of the TCGA according (**A**) to their pN (*LN: lymph node*) status (*n* = 867; *p* < 0.001), (**B**) to their pT (tumor size) status (*n* = 763; n.s.), (**C**) to their tumor grading (*n* = 863; n.s.), (**D**) to their age and menopausal (*prem: premenopausal; postm: postmenopausal*) status (*n* = 696; n.s.), (**E**) to their histological subtype (*n* = 829; n.s.), and (**F**) to their Her2 expression status (*n* = 742; n.s.). *Boxplots:* *Whiskers represent the 10th and the 90th percentile, crosses represent outliers, and the red line marks the mean* value. *Number of samples analysed is given in brackets above each box. Wilcoxon rank sum* tests *were used to compare two groups and Kruskal Wallis* test*s with Dunn's post-hoc* test*s for pairwise comparison were used to compare more than two groups. n.c.: normalized counts; (*−*): negative; (+): positive; n.s.: not significant.*

**Figure 3:**
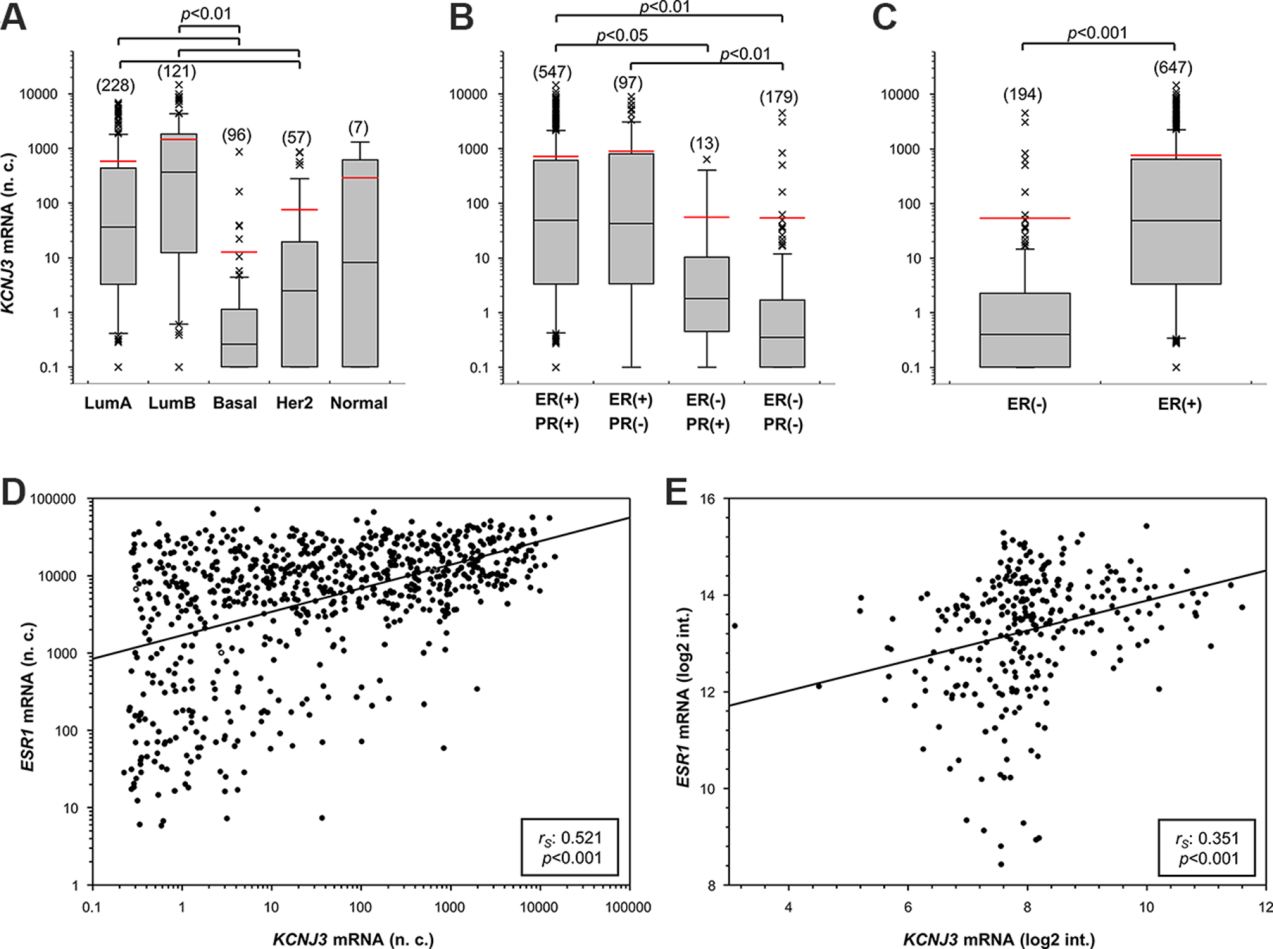
*KCNJ3* mRNA levels are upregulated in estrogen receptor positive breast cancer samples *KCNJ3* mRNA expression levels in breast cancer patient samples of the TCGA according (**A**) to their PAM50 classification (*n* = 509), (**B**) to their estrogen and progesterone receptor status (*n* = 836), and (**C**) to their estrogen receptor status (*n* = 841; *p* < 0.001). *Boxplots:* *Whiskers represent the 10th and the 90th percentile, crosses represent outliers, and the red line marks the mean* value*. Number of samples analysed is given in brackets above each box. A Kruskal Wallis* test *with Dunn's post-hoc* test*s for pairwise comparison was used for analysis in (A) and (B), and a Wilcoxon rank sum* test *in (C); p*-value*s are given on top of each plot.* (**D**) Scatter plot of *KCNJ3* and *ESR1* mRNA expression levels in breast cancer patient samples of the TCGA (*n* = 905). (**E**) Scatter plot of *KCNJ3* and *ESR1* mRNA expression levels in patient samples of the GEO data set GSE17705 (*n* = 298). *Spearman rank correlation coefficients (r*_S_*) and p*-value*s are given in the boxes of each plot. n.c.: normalized counts; log2 int.: log2 intensity; LumA: luminal A type; LumB: luminal B type; basal: basal type; Her2: Her2-enriched type; Normal: normal-like type; ER: estrogen receptor; PR: progesterone receptor; (*−*): negative; (+): positive.*

Next, we studied *KCNJ3* mRNA expression levels of the TCGA data set to detect possible associations with clinically relevant breast cancer subsets. We observed increased *KCNJ3* expression in tumors of patients who presented with positive lymph nodes when compared to those without lymph node metastasis at diagnosis (median 6.9 vs. 25.3 normalized counts; *p* < 0.001; Figure 2A). *KCNJ3* mRNA levels did not differ significantly when patients were grouped based on their tumor size, tumor grade, age, menopausal status, histological subtype or Her2 expression status (Figure 2B–2F). Then, patients were categorized according to their PAM50 classification (luminal A, luminal B, Her2-enriched, basal-like, normal-like): patients of both the luminal A and B subtype had significantly higher *KCNJ3* expression levels than patients of the basal or Her2-enriched type (*p* < 0.001; Figure 3A). No relevant information could be retrieved from the normal-like subtype due to the low patient number (*n* = 7). Since the luminal A and B subtypes include the hormone receptor positive tumors, we analyzed the patients regarding their estrogen and progesterone receptor (ER and PR) status. The two ER positive groups (ER+/PR+ and ER+/PR−) had significantly higher *KCNJ3* levels than those that were negative for ER and PR (*p* < 0.001; Figure 3B). Hence we conclude that high *KCNJ3* expression levels are associated with positive ER, but not PR status. Finally, comparison of patients grouped solely based on ER status revealed substantially higher *KCNJ3* levels in ER positive patients (median 0.4 vs. 48.9 normalized counts; *p* < 0.001; Figure 3C).

Based on these findings, we investigated whether *KCNJ3* expression would correlate with the gene expression levels of the three known estrogen receptors *ESR1* (ERα,), *ESR2* (ERβ) and *GPER* (G-protein coupled ER 1; synonym: GPR30). Spearman rank correlation analysis showed that *KCNJ3* expression correlates with *ESR1* (*r*_S_: 0.521; *p* < 0.001; Figure 3D) but not with the other two estrogen receptors *ESR2* (*r*_S_: −0.191) and *GPER* (*r*_S_: 0.253). In light of this finding, it was of interest to examine the correlation between *KCNJ3* and *ESR1* expression in our validation data set (GEO ID GSE17705). A positive correlation between *KCNJ3* and *ESR1* expression was observed (*r*_S_: 0.351; *p* < 0.001; Figure 3E), but not between *KCNJ3* and *ESR2* (*r*_S_: 0.165) or *GPER* (*r*_S:_ 0.09).

**Figure 4:**
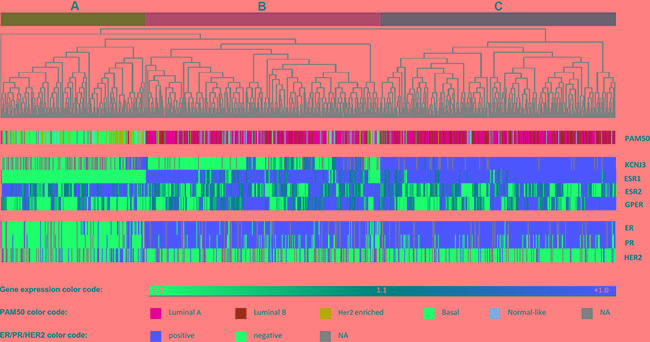
Heatmap of hierarchical cluster analysis Hierarchical cluster analysis of mRNA levels of *KCNJ3* and the estrogen receptors α *(ESR1)*, β *(ESR2)*, and GPR30 *(GPER)*. The gene expression relative to the median expression value across all samples is shown (blue: values below median; red: values above median). PAM50 classification (multicolor panel) and pathological ER, PR and Her2 expression status (blue: negative; red: positive) are given for each patient sample. The dendrogram on top shows that patients were divided into three main branches (**A**, **B** and **C**) based on the expression levels of the four genes analyzed.

Furthermore, we performed hierarchical cluster analysis with *KCNJ3*, *ESR1*, *ESR2* and *GPER* (Figure 4). Based on the expression levels of those four genes, patient samples clustered in three major groups designated A, B and C in Figure 4. Group A, which showed low *KCNJ3* expression levels, comprised patients of the basal and the Her2-enriched PAM50 type, being generally negative for pathological ER and PR status and displaying low *ESR1* expression levels. Groups B and C were mainly composed of patients of the luminal A and B types, being positive for ER and PR status and displaying high *ESR1* expression. The major difference between group B and C was the *KCNJ3* expression (low in group B and high in group C; Figure 4). Further, it became evident that, again, *KCNJ3* expression clustered with *ESR1*, but not with *ESR2* and *GPER.*

### *KCNJ3* is an independent prognostic factor in ER positive patients

**Figure 5:**
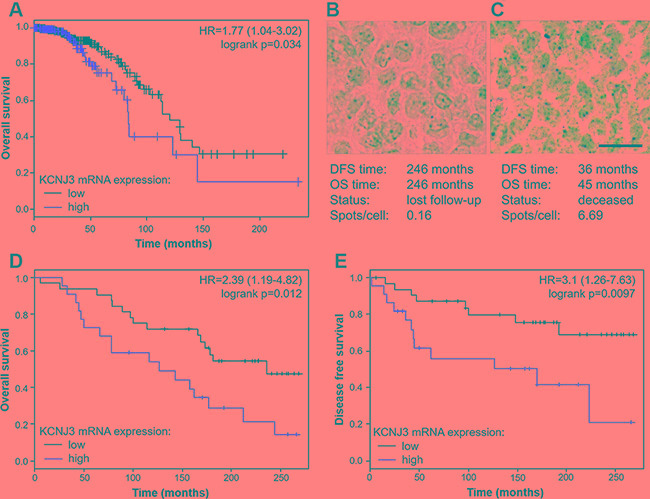
Estrogen receptor positive patients with high *KCNJ3* mRNA levels in their tumor have a worse overall and disease free survival probability (**A**) Kaplan-Meier plot showing overall survival of ER positive patients of the TCGA data set with low and high *KCNJ3* mRNA expression levels as determined by RNAseq (*n* = 647). (**B**) Representative RNA *in situ* hybridization of a patient with low *KCNJ3* levels in the tumor (0.16 spots/cell) and long survival times. (**C**) Representative RNA *in situ* hybridization of a patient with high *KCNJ3* levels in the tumor (6.69 spots/cell) and short survival times. (**D**) Kaplan-Meier plot showing overall survival of a subset of patients of the GEO data set GSE17705 with low and high *KCNJ3* mRNA expression levels as determined by RNA *in situ* hybridization (*n* = 54). (**E**) Same as (D), but showing disease free survival. *Hazard ratios and logrank p*-value*s are given in Kaplan-Meier plots. Scale bar in (B) and (C): 20μm. DFS: disease free survival; OS: overall survival.*

Based on the results demonstrated above, we further focused on ER positive patients (groups B and C in Figure 4). Overall survival analysis of ER positive patients of the TCGA data set revealed that those with high *KCNJ3* levels in the tumor (group C) had shorter overall survival times than patients of group B with low *KCNJ3* levels (*n* = 647; *p* < 0.05; HR = 1.77 (1.04–3.02); Figure 5A). In addition, we used a Cox-proportional hazard approach for univariate and multivariate survival analysis of estrogen receptor positive patients (Table 1). The univariate Cox model showed that age at diagnosis as well as lymph node, metastasis, PAM50 and *KCNJ3* status had a significant influence on survival times. Importantly, the multivariate Cox model − with tumor size, lymph node status, metastasis status, histology, Her2 status, menopause status, age at diagnosis, PAM50 classification and *KCNJ3* status as co-variates − showed that only the *KCNJ3* expression status had a significant impact on survival time (*n* = 228; *p* < 0.05; HR = 5.2 (1.3–21.8); Table 1).

**Table 1:** Cox-proportional hazard models for estrogen receptor positive breast cancer patients

		univariate Cox model^a^		multivariate Cox model^b^		multivariate Cox model^c^	
		HR (95%-CI)	*p*-value	HR(95%-CI)	*p*-value	HR(95%-CI)	*p*-value
**Size (T)**	T1 vs. T2|T3|T4	1.0 (0.6–1.8)	0.985	4.2 (0.5–36.2)	0.184	5.9 (0.7–53.9)	0.113
**Lymph node status (N)**	neg. vs. pos.	**1.8 (1.0–3.2)**	**0.042**	0.7 (0.2–2.4)	0.612	0.7 (0.2–2.2)	0.513
**Metastasis status (M)**	neg.vs.pos.	**3.5 (1.5–8.3)**	**0.004**	3.9 (0.7–21.4)	0.112	4.3 (0.8–23.2)	0.092
**Histology**	ductal vs. lobular	0.6 (0.1–2.4)	0.454	0.6 (0.1–3.2)	0.538	0.5(0.1–3.3)	0.539
**Her2 status**	neg. vs. pos.	1.1 (0.4–2.8)	0.845	1.2 (0.1–10.3)	0.894	2.2 (0.2–22.5)	0.517
**Menopause status**	prem. vs. postm.	2.5 (0.9–7.0)	0.092	0.9(0.04–18.4)	0.928	0.6(0.02–20.9)	0.753
**Age at diagnosis**	≤50 vs. > 50 years	**1.9 (1.0–3.4)**	**0.038**	3.2 (0.9–11.1)	0.812	2.7(0.1–116.0)	0.589
**PAM50 status**	Luminal A vs. Luminal B	**2.6 (1.3–5.3)**	**0.006**	3.2 (0.1–32.5)	0.071	1.5(0.4–5.9)	0.589
**KCNJ3 expression**	low(≤ 72th %ile) vs. high (> 72th %ile)	**1.8(1.0–3.0)**	**0.036**			**5.2(1.3–21.8)**	**0.021**

a *n* = 646

b without *KCNJ3* mRNA expression, *n* = 228

c with *KCNJ3* mRNA expression, *n* = 228

Abbreviations: neg.: negative; pos.: positive; prem.: premenopausal; postm.: postmenopausal; HR: hazard ratio; CI: confidence interval

To validate our findings and to extend our analysis beyond the TCGA data set, we performed *KCNJ3* RNA *in situ* hybridization (ISH) on 66 breast cancer patient samples that were available with clinical data and follow-up times from the estrogen receptor positive cohort GSE17705. Patient characteristics of this cohort compared to the TCGA cohort are shown in Table 2. RNA ISH was the method of choice because we previously showed superiority of ISH over classical immunohistochemistry regarding sensitivity and specificity [18]. Twelve of the samples analyzed did not meet the quality control criteria for ISH (see methods section) and were therefore excluded from further analysis (remaining *n* = 54). Representative images of patient samples with either low *KCNJ3* levels and long survival times or high *KCNJ3* levels and short survival times are given in Figure 5B and 5C. Kaplan Meier analyses showed significantly shorter overall survival (*p* < 0.05; HR = 2.39 (1.19–4.82); Figure 5D) and disease free survival (*p* < 0.01; HR = 3.1 (1.26–7.63); Figure 5E) probabilities for patients with high *KCNJ3* expression.

### MCF-7 cells overexpressing *KCNJ3* display higher self-renewal capacity

**Figure 6:**
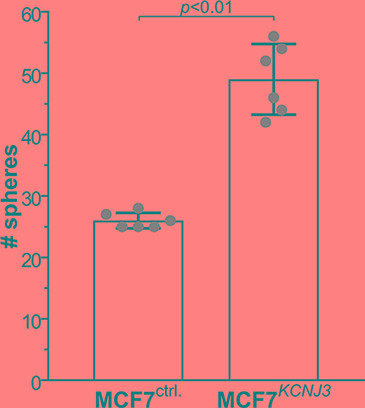
MCF-7 cells overexpressing *KCNJ3* form more mammospheres than controls Mammosphere formation of MCF-7 control cells (overexpressing empty vector; MCF-7^ctrl.^) and MCF-7 cells overexpressing *KCNJ3* (MCF-7^*KCNJ3*^). *Single data points are shown by grey circles. # spheres: number of mammospheres counted (mean ± standard deviation). A Wilcoxon rank sum* test *was used for analysis.*

In order to gain additional insight into the cellular mechanisms that would lead to worse patient outcomes due to high levels of *KCNJ3* in tumor cells, we performed a mammosphere formation assay with the ER positive breast cancer cell line MCF-7. This was of particular interest, as pathological upregulation of different potassium channels has been shown to affect cancer stem cell properties [19–21], and sphere formation is linked to self-renewal capacity and stemness of cancer cells. MCF-7 cells stably overexpressing *KCNJ3* (MCF-7^GIRK1a^; [13]) formed significantly higher numbers of mammospheres compared to controls (*p* < 0.01; Figure 6), indicating that *KCNJ3* upregulation might be involved in conferring self-renewal capacity to cancer cells and thus contributing to higher tumor aggressiveness.

## DISCUSSION

We have analyzed > 1000 breast cancer patient samples of two independent data sets (TCGA: *n* = 905, GSE17705: *n* = 298) regarding their *KCNJ3* expression in order to evaluate a potential prognostic role of this ion channel gene for breast cancer.

Generally, ion channels have gained increased attention as players in cancer development and metastasis, since aberrant expression of as well as mutations in several genes encoding ion channels have been found to influence the hallmarks of cancer towards higher malignancy [22–26]. Potassium channels have been most comprehensively studied regarding their oncogenic potential by promoting proliferation and apoptosis [27–32]. Several studies suggested evidence for a role of GIRK1, the G-protein coupled inward rectifier K+ channel encoded by *KCNJ3*, in breast cancer [10–12]. Our results underscore these seminal findings using a substantially larger number of patient samples: we observed a significant increase of *KCNJ3* mRNA expression levels in tumors of lymph node positive patients when compared to lymph node negative ones in our evaluation of 867 patients, and *KCNJ3* mRNA levels were significantly higher in breast cancer samples than in corresponding normal breast tissue in a set of 105 patient samples.

The positive correlation between *KCNJ3* levels and the ER status of breast cancer samples shown here had not been detected previously by the studies of Brevet et al. and Stringer et al. [11, 12], supposedly because of the sizes of their patient cohorts (*n* = 56 and *n* = 31, respectively). In contrast, Ko et al. [33] performed ion channel profiling in breast cancer and mentioned a decrease of *KCNJ3* expression in p53 mutant breast tumors, which are more likely to be ER negative [34], and an increase in *KCNJ3* mRNA expression in ER positive tumors when compared to ER negative samples. However, no further details or survival data regarding *KCNJ3* expression were given or discussed, as this study aimed to develop an ion channel gene signature (termed IC30) as prognostic tool in breast cancer, and *KCNJ3* is not comprised in this final gene panel [33]. In line with this, our data clearly demonstrate that *KCNJ3* expression is associated with ER positive breast cancer and that *KCNJ3* levels correlate with *ESR1* mRNA expression levels but not with expression levels of other estrogen receptors. However, *KCNJ3* levels were variable within the ER positive patient cohorts. Most importantly, we could show in two independent data sets and by two different methods (gene expression data and RNA *in situ* hybridization) that ER positive patients with high *KCNJ3* expression levels had worse overall and disease free survival probabilities than ER positive patients with low *KCNJ3* levels. This is further corroborated by multivariate Cox proportional hazard analysis, showing that *KCNJ3* is an independent prognostic marker for ER positive breast cancer, being also independent from the PAM50 subtype of the patient. Therefore, ER positive patients might be stratified into high risk and low risk groups based on the *KCNJ3* levels in the tumor.

Here, we used a highly sensitive and specific RNA *in situ* hybridization technique for the validation of our findings, that showed positive signals in tumor cells but not in peritumoral tissue, and that allowed to clearly discriminate between patients with low and high *KCNJ3* expression. It might be argued that immunohistochemistry would be the method of choice when investigating novel tumor markers. However, and several tested anti-GIRK1 antibodies did not meet the high quality standards regarding sensitivity and specificity that are required for optimal results in immunohistochemistry. Based on a comparison of the two methods, we came to the conclusion that RNA ISH is the superior technique for studying *KCNJ3* expression in tissue [18].

To date, the mechanisms leading to *KCNJ3* upregulation in breast carcinomas are not understood. There is no evidence for estrogen response elements (ERE) in the promoter region of *KCNJ3* [35, 36] making a direct activation of *KCNJ3* gene transcription via ER unlikely to occur. Our results also argue against amplification of the *KCNJ3* gene locus as the underlying mechanism for *KCNJ3* upregulation. Further, it is unknown how the GIRK1 protein might interact with the estrogen receptor. GIRK1 is a non-functional channel when expressed as a homomer [37], located intracellularly as it accumulates in the endoplasmatic reticulum upon overexpression [38]. Thus, it remains to be determined whether and how GIRK1 and ER act together in same signaling pathways and further studies are needed to elucidate the mechanism of action of *KCNJ3* upregulation in breast cancer.

On a more functional level, our research group provided evidence on downstream effects of *KCNJ3* upregulation in breast cancer: Rezania et al. showed that MCF-7 cells display higher wound healing capacity, increased invasion towards chemoattractants and higher motility and velocity than controls upon stable overexpression of *KCNJ3* [13]. We could further corroborate these findings by showing that MCF-7 cells stably overexpressing *KCNJ3* display significantly higher self-renewal capacity. Taken together, we propose a role of *KCNJ3* in conferring tumor aggressiveness via invasion, metastasis and increased self-renewal capacity.

In conclusion, the present study a) confirms in two independent data sets, that *KCNJ3* is upregulated in breast carcinomas when compared to normal breast tissue, b) shows that increased *KCNJ3* expression is significantly associated with estrogen receptor positive breast cancer subtypes, c) highlights that increased *KCNJ3* is an independent prognostic marker conferring worse overall and disease free survival probabilities to estrogen receptor positive breast cancer patients, and d) demonstrates that *KCNJ3* upregulation might be involved in conferring higher self-renewal capacity to cancer cells. Future studies are required to elucidate the mechanisms that lead to *KCNJ3* upregulation in ER positive breast cancer, to unveil its involvement in invasion and metastasis and to evaluate its potential as drugable target.

## MATERIALS AND METHODS

### Gene expression data from the cancer genome atlas

The gene expression levels (RNAseq V2 level 3 data) of 950 invasive breast carcinoma samples and of 105 samples from corresponding healthy tissues were downloaded from the The Cancer Genome Atlas (TCGA) data portal (https://tcga-data.nci.nih.gov) and the upper quartile normalized counts from the RSEM pipeline were used. The corresponding clinical data of each patient were downloaded from the University of California Santa Cruz (UCSC) Cancer Genomics Browser (https://genome-cancer.ucsc.edu). Male patients (*n* = 9) and patients without gene expression data for *KCNJ3* (*n* = 36) were excluded for further analysis (remaining *n* = 905). Patient characteristics are summarized in Table 2.

**Table 2:** Patient characteristics of the two data sets studied

	TCGA data set (%)(*n* = 905)	Validation set (%)(*n* = 66)	*p*-value^a^
**Age at diagnosis (years)**			<0.001
≤ 50	26.6	10.6	
> 50	61.0	89.4	
n.a.	12.4	0	
mean ± SD	58 ± 13	63.7 ± 9.8	
**Status**			< 0.001
alive/lost for follow-up	80.2	42.4	
deceased	10.8	57.6	
n.a.	9.0	0	
**Follow-up time (months)**			< 0.001
mean ± SD	32 ± 35	157 ± 76	
Lymph node status			n.s.
negative	46.1	51.5	
positive	49.7	45.5	
n.a.	4.2	3.0	
**Metastasis status**			n.s.
negative	86.4	100	
positive	1.7	0	
n.a.	11.9	0	
**T stage**			n.s.
pT1	26.0	40.9	
pT2	56.2	48.5	
pT3	11.3	6.1	
pT4	3.6	4.5	
n.a.	2.9	0	
**Grade**			n.s.
1	16.6	18.2	
2	55.8	54.6	
3	21.4	24.2	
4	1.7	0	
n.a.	4.5	3.0	
**ER status**			< 0.001
negative	21.4	0	
positive	71.5	100	
n.a.	7.1	0	
**PR status**			n.s.
negative	30.6	24.2	
positive	62.0	75.8	
n.a.	7.4	0	
**Her2 status**			n.a.
negative	70.2		
positive	11.8		
n.a.	18.0	100	
**PAM50 classification**			n.a.
luminal A	25.2		
luminal B	13.4		
Her-2 enriched	6.3		
basal-like	10.6		
normal-like	0.8		
n.a.	43.6	100	

a A Mann-Whitney rank sum test was used to compare the groups regarding age at diagnosis and follow-up time; and a chi-square test was used to compare the groups regarding all other characteristics.

Abbreviations: SD: standard deviation; n.s.: not significant; not available/applicable; ER: estrogen receptor; PR: progesterone receptor.

### Gene expression data from GEO ID GSE17705

The normalized gene expression levels of the estrogen receptor positive cohort GSE17705 (*n* = 298) were downloaded from the Gene Expression Omnibus (GEO). The data were generated and processed using the Affymetrix Human Genome U133A Array platform as described [14]. Briefly, tissue samples were processed by two different laboratories (MD Anderson Cancer Center, Texas, USA and Jules Bordet Institute, Brussels, Belgium; inter-laboratory reliability was assessed) and probe-level intensities were generated with Microarray Suite (MAS) version 5.0, normalized and log2 transformed.

### FFPE patient samples

Detailed corresponding clinical and follow-up data as well as formalin-fixed, paraffin-embedded (FFPE) tissue samples were available for 66 patients of the ER positive cohort GSE17705. FFPE samples were collected from the Medical University of Graz, Austria and the Medical University of Innsbruck, Austria (see Table 2 for patient characteristics) [14]. Differences between the two data sets are given by a) the estrogen receptor status (all positive in the validation cohort, mixed in the TCGA) and b) the age, follow-up time and status which are more detailed and complete in the validation cohort than in the TCGA data set. The use of the patient samples including the clinical data was approved by the ethics committee of the Medical University of Graz (24-081 ex 11/12).

### RNA *in-situ* hybridization

FFPE tissue sections (thickness 4 μm) were mounted on Superfrost Plus coated slides (Thermo Scientific, Waltham, MA, USA; #10135642) and peritumoral tissue devoid of cancer was trimmed. Slides were treated according to manufacturer's instructions for the RNAscope^®^ 2.0 High Definition - BROWN kit (ACD, Hayward, CA, USA; #310035). Briefly, the slides were incubated at 60 °C for 1 hour followed by pretreatment (pretreat 1: 10 min, pretreat 2: 15 min, pretreat 3: 30 min). Three sections of each sample were stained with different probes: the *KCNJ3* probe (#Hs-KCNJ3-tv1tv2), the negative control probe bacterial dihydrodipicolinate reductase (DapB; #310043), and the positive control probe DNA-directed RNA polymerase II subunit RPB1 (POLR2A; #310451). Probes were incubated for 2 hours at 40°C using the HybEZ oven from ACD and six signal amplification steps, followed by signal detection with DAB were performed according to manufacturer's protocol. Slides were counterstained with 50% hematoxylin, dehydrated, and cover slipped.

### Image analysis

Slides were first assessed by microscopic inspection. Since the staining pattern of RNA *in situ* hybridization was homogenous across different regions of the same sample, a representative region was selected for each tumor, and z-stacks comprising 10 images were captured at 40× magnification using a Zeiss Observer.Z1 inverted microscope (Zeiss, Jena, Germany). Multiple adjacent single images (3x3 tiles) were acquired and aligned using the MosaiX module of the AxioVision software (Zeiss). Each assembled image covered an area of approx. 0.62 × 0.45 mm (3981x2980 pixels). Image sequences were stacked using the minimal intensity projection type setting of the ImageJ software (http://imagej.nih.gov/ij/). The SpotStudio software from ACD was used for detection of single cells, detection of spots and clusters and calculation of the estimated number of spots per cell. *DapB* and *POLR2A* probes served as technical quality controls that needed to fulfill the below given cut-off criteria in order to a) ensure technical specificity of the probes (negative control) and b) to detect samples with highly degraded RNA (positive control). The maximum threshold for negative controls was set at 0.5 spots/cell and the minimum threshold for positive controls at 2.5 spots/cell. Samples not fulfilling these cut-off requirements were excluded from further analysis.

### Cell culture

The breast cancer cell line MCF-7 (ATCC; Manassas, VA, USA; #HTB-22™) was grown in minimal essential medium (Gibco, life technology, Grand Island, NY, USA; #31095_029) supplemented with 10% fetal bovine serum (Sigma Aldrich, St. Louis, USA; #P2442), penicillin/streptomycin (100 U/mL and 100 ng/mL; Sigma Aldrich; #P0781), and 1mmol/L sodium pyruvate (Sigma Aldrich; #S8636). MCF-7 cells stably overexpressing *KCNJ3* were produced and cultured as described [13]. Cells were kept in a humidified atmosphere at 37°C and 5% CO_2_. Mycoplasma tests were negative, and short tandem repeat (STR) profiling proofed the cell line to be authentic.

### Mammosphere formation assay

The mammosphere formation assay was performed as described previously [39] to test the self-renewal capacity of MCF-7 cells overexpressing *KCNJ3*. Briefly, MCF-7 cells were harvested and seeded in ultra-low attachment 6-well plates (2000 cells/well; Corning; NY, USA; #3471) with serum-free medium (MEBM; Lonza; Basel, Switzerland; #CC3151) supplemented with 1xB27 (Gibco; #17504044), 20 ng/ml human epidermal growth factor (Peprotech; Hamburg, Germany; #AF-100-15), 10 ng/ml human basic fibroblast growth factor (Peprotech; #AF-100-18C), 20 IU/ml Heparin (Baxter; Vienna, Austria) and 1% antibiotic/antimycotic solution (Sigma-Aldrich; #A5955). Mammospheres were observed and counted after 10 days.

### Statistical analysis

Statistical analyses were performed using the SigmaPlot/SigmaStat v12.5 software (Systat Software Inc., San Jose, CA, USA) or GraphPad Prism 7.02 for Windows (GraphPad Software, La Jolla, CA, USA) for comparison of groups, Spearman rank correlation analysis, generation of boxplots, bargraphs and scatter plots. All performed tests were two-sided and rank based. For comparisons of paired data, a Wilcoxon signed rank test, for comparisons of two groups a Wilcoxon rank sum test (Mann-Whitney U test), and for comparison for more than two groups a Kruskal-Wallis test followed by Dunn's posthoc tests for pairwise comparisons were used. Different patient characteristics between the TCGA cohort and the validation cohort (subset of GEO ID GSE17705) were compared using a chi-square test and a Wilcoxon rank sum test regarding age at diagnosis and follow-up times. Genesis 1.7.6 (Graz University of Technology, Graz, Austria) was used for log2-transformation and mean centering of gene expression values, calculation of Euclidean distance, hierarchical cluster analysis and heatmap visualization. For overall and disease free survival analysis, the statistical software environment R (www.r-project.org) including the package *survival* and an adopted code for the auto-cut-off option from Györffy et al. [40] was used. For the construction of survival curves, a Kaplan-Meier estimator was used and survival curves of patient groups with high expression versus low expression were compared by a logrank test. A time-independent Cox-proportional hazard approach was applied for univariate and multivariate survival analysis. Results were considered statistically significant when *p* < 0.05.

### In alphabethical order

DFS : disease free survival, ER : estrogen receptor, FFPE : formalin-fixed, paraffin-embedded, GEO : Gene Expression, Omnibus, GIRK : G-protein activated, inward rectifier potassium channel, HR : hazard ratio, ISH : *in situ* hybridization, LN : lymph node, log_2_ int. : log_2_ intensity, LumA : luminal A, LumB : luminal B, n.c. : normalized counts, OS : overall survival, PR : progesterone receptor, *r_S_ :* Spearman rank, correlation coefficient, TCGA :The Cancer Genome Atlas.
